# Kinect and wearable inertial sensors for motor rehabilitation programs at home: state of the art and an experimental comparison

**DOI:** 10.1186/s12938-020-00762-7

**Published:** 2020-04-23

**Authors:** Bojan Milosevic, Alberto Leardini, Elisabetta Farella

**Affiliations:** 1 E3DA, Fondazione Bruno Kessler (FBK), Trento, Italy; 2 Movement Analysis Laboratory, IRCCS Istituto Ortopedico Rizzoli, Bologna, Italy

**Keywords:** Motor rehabilitation, Home rehabilitation, wearable inertial sensors, Kinect

## Abstract

**Background:**

Emerging sensing and communication technologies are contributing to the development of many motor rehabilitation programs outside the standard healthcare facilities. Nowadays, motor rehabilitation exercises can be easily performed and monitored even at home by a variety of motion-tracking systems. These are cheap, reliable, easy-to-use, and allow also remote configuration and control of the rehabilitation programs. The two most promising technologies for home-based motor rehabilitation programs are inertial wearable sensors and video-based motion capture systems.

**Methods:**

In this paper, after a thorough review of the relevant literature, an original experimental analysis is reported for two corresponding commercially available solutions, a wearable inertial measurement unit and the Kinect, respectively. For the former, a number of different algorithms for rigid body pose estimation from sensor data were also tested. Both systems were compared with the measurements obtained with state-of-the-art marker-based stereophotogrammetric motion analysis, taken as a gold-standard, and also evaluated outside the lab in a home environment.

**Results:**

The results in the laboratory setting showed similarly good performance for the elementary large motion exercises, with both systems having errors in the 3–8 degree range. Usability and other possible limitations were also assessed during utilization at home, which revealed additional advantages and drawbacks for the two systems.

**Conclusions:**

The two evaluated systems use different technology and algorithms, but have similar performance in terms of human motion tracking. Therefore, both can be adopted for monitoring home-based rehabilitation programs, taking adequate precautions however for operation, user instructions and interpretation of the results.

## Background

Emerging sensing and communication technologies are driving the innovation of a vast number of application fields, including fitness, healthcare and rehabilitation therapy [1]. Major drivers of healthcare innovation include the priority changes from treatment to prevention, and the search to provide personalized and patient-centric solutions. Both trends are enabled by unobtrusive sensing technologies, allowing for continuous monitoring and increased engagement with the patient outside the clinic [2]. Movement analysis and its use for motor rehabilitation is one of the many application fields where innovative technical solutions for unconstrained and autonomous monitoring of the patients are being adopted [3].

Standard practices for motor rehabilitation include the clinician’s supervision and evaluation of the patient’s movements, when performed during therapy sessions in clinic, and no supervision or any feedback when the exercises are executed at home. Computer vision and stereophotogrammetry-based technologies have been widely proven as accurate and reliable tools for objective measurement of human motion [4, 5]. However, the costs and difficulties of operation of such systems have limited their use to research rather than in everyday clinical and rehabilitation practice. The development of miniaturized inertial sensors paved the way for the development of wearable Inertial Measurement Units (IMUs) and their use for motion capture [6, 7]. Such technologies have also been validated in lab environments for medical applications and motor rehabilitation analyses [8, 9]; however, the available solutions involve cost and complexity-related limitations.

Nowadays, both research and commercial applications are experiencing a push in ubiquitous computing and the use of wearable and interconnected sensing devices for a wide range of applications, from entertainment to fitness and wellbeing [10]. The adoption of the use of fitness and activity trackers is driven by their low cost and ease of use, but these have usually limited accuracy in the reported data [11]. For a successful adoption of these new technologies in rehabilitation, there is a need to evaluate their accuracy and reliability and to provide insights on their proper use in order to define best practices and standardized protocols [12]. The recent innovative low-cost sensing solutions and relevant algorithms for data analysis, once validated, can be effectively introduced in rehabilitation protocols both in specialized centers and at home, and truly enable a patient-centric, preventive and smart healthcare revolution [13].

In the field of human motion analysis, both video and inertial-based solutions have now low-cost options, suitable for wide adoption and everyday use; examples include the Kinect camera [14] and various activity tracking and wearable inertial sensors [15]. Their integration into bio-feedback-based systems and combination with exergames and appropriate back-end infrastructure allows for the development of innovative solutions for real-time monitoring of home-based rehabilitation therapies and for a continuous remote supervision by the clinician [16]. The first platforms providing such functionalities include DoctorKinetic (DoctorKinetic, Netherlands), SilverFit (SilverFit, Netherlands) and Riablo (Corehab, Italy).

This paper reports an overview of these major systems, analyzing in the literature the state-of-the-art of the Kinect and of wearable motion sensing in rehabilitation, but mainly focuses on a validation work for the quantitative assessment of these systems. Since there is a lack of direct comparison and discussion on the differences of the two technologies, an original experimental study was performed and here reported to evaluate and directly compare the Kinect v2 and a commercially available wearable IMU (EXLs3 by Exel srl, Italy). Their technological characteristics and state-of-the-art algorithms for IMU-based analysis were assessed, using a marker-based stereophotogrammetry motion analysis system as the gold standard. In addition to laboratory tests, the two systems were also assessed in a typical home environment, to evaluate and fully compare their final usability and robustness. The two systems are here compared based on exactly the same human motion exercises, both in a laboratory setting for a thorough comparison with state-of-the-art motion analysis, and at home, for simulation of final users’ conditions. Finally, drawing from the current state-of-the-art and from the present experimental comparisons, the main advantages and disadvantages of the two systems are discussed, analyzing their strengths and weaknesses, and highlighting the challenges for their successful future adoption in the rehabilitation context.

## Review of sensing technologies for motion analysis in rehabilitation

This section will analyze current systems in human motion analysis, starting from the well-established video motion capture used in gait laboratories, and then focusing on innovative and low-cost alternatives, suitable for autonomous use at home. The reported references are summarized and compared in Table 1.

**Table 1:** Summary of the main validation studies analyzing motion capture accuracy of the Kinect (v1 and v2) and of wearable IMUs

Refs.	Sensor(s)	Participants	Exercises	Parameters	Applications	Performance
[49]	Kinect v1	1H	6 primary movements	Joints positions, bone lengths	At-home elderly rehabilitation	RMSE $< 10cm$
[50]	Kinect v1	20H	Reaching, standing balance	Joints positions	Postural control	ICC $< 0.16$ CV $<11.6\%$
[51]	Kinect v1	48H, 2 sessions	4 primary movements	Joints angles	Funct. assessment	RMSE $<9\%$
[52]	Kinect v1	10H + 9PD	12 primary and functional movements	Joints positions and angles, timings	PD assessment	LoA $<10\%$ ICC $>0.9$
[53]	Kinect v1	20H	Standing sway	Marker positions	Exergaming	SDev $<30\%$
[54]	Kinect v1	15H	Squat	Lower limb joints angles	Kinect evaluation	RMSE $<5^\circ$ ICC $>0.9$
[36]	Kinect v2	30H, 2 sessions	Standing balance	Trunk and pelvis angles	Balance and postural control	r $>0.75$ ICC $>0.7$
[42]	Kinect v2	30H, 2 sessions	Walking	Lower limbs joints angles	Gait analysis	$0.4<$ r $< 0.75$
[39]	Kinect v2	20H	5 primary shoulder movements	Upper limbs joints angles	Clinic and home rehabilitation	RMSE = $3.9^\circ$
[43]	Kinect v2	20H	Walking	Stance and step time, step length, time, velocity	Gait analysis	LoA $<11\%$ ICC $>0.9$
[41]	Kinect v2	30H	Standing and sitting exercises	Upper body joint positions	Rehabilitation	RMSE $<5\%$ r $>0.9$
[55]	4 IMU (custom) on thighs, shanks	3H	Walking	Hip and knee joint angles	Gait analysis	RMSE $<8.7^\circ$ r $>0.7$
[6]	2 IMU (Xsens MTx) on pelvis, thigh	20H	Walking	Hip joint angle	Gait analysis	LoA $= 1.6^\circ$
[56]	8 IMU (Xsens MTx) on thorax, lower back, thighs, shanks, feet	9H (children)	Static postures	Hip and knee joint angles	Knee amputees and cerebral palsy	RMSE $=1.8^\circ$
[57]	6 IMU (not specified) on pelvis, thigh, shank	9H + 1INJ	Running and outdoor training routines	Activity classification, hip and knee joints angles	Athlete training, injury prevention	98%
[58]	3 IMU (Shimmer) on thigh, shank, foot	58REH	Primary movements	Exercise recognition	Clinic and home rehabilitation	83%
[8]	5 IMU (Riablo) on trunk, thigh, shank	22H, 2 sessions	4 knee exercises	Hip and knee joint angles	Clinic and home rehabilitation	RMSE $=3.1^\circ$
[59]	2 IMU (Opal) on lower back, upper arm	6H	ADL routines, walking	Trunk and upper limb joints angles	IMU evaluation	RMSE $<5.5^\circ$
[60]	6 IMU (Xsens MTx) on thigh, shank, foot	1AMP	Walking	Knee angle	Gait analysis	RMSE $<3^\circ$
[61]	7 IMU (Opal) on trunk, upper arm, forearm, hand	8H	2 swimming styles	Upper limb joints angles	swimming analysis	RMSE $<7^\circ$
[62]	3 IMU (Opal) on waist, thigh, shank, 1 resistive strip	14H	Sit-to-stand, walking	Exercise timings, stride length	Clinic and home rehabilitation	r $>0.7$ ICC $>0.95$
[63]	17 IMU (Synertial) full body	20H	5m timed up and go	Full body joints	Clinic evaluation	RMSE $<13.6^\circ$
[64]	2 IMU (APDM) thigh and shank	18REH	Knee ROM, walking	Knee angle	Clinic rehabilitation	–
[9]	17 IMU (Xsens MVN) full body	12H	Functional movements, work actions	Full body model	Occupational biomechanics	RMSE $<5^\circ$
[65]	3 IMU (I2M MT) on pelvis, trunk, upper arm	6H lab + 10H workplace	Work actions	Trunk orientation, shoulder joint angles	Occupational biomechanics	RMSE $<12.1^\circ$
[47]	Kinect v1, Kinect v2	20H (v1) + 20H (v2)	15 static poses	Joints positions	Posture evaluation	RMSE(v1) $=76mm$ RMSE(v2) $=87mm$
[48]	Kinect v1, Kinect v2	10H, 3 camera angles	Sitting and standing postures	Joints positions	Motion capture	RMSE $<100mm$
[33]	Kinect v1, Kinect v2	13H	ROM and static stretching exercises	Joints positions	Rehabilitation	RMSE $=15mm$(wrist) RMSE $<10mm$(other)
[66]	Kinect v1 + 2IMU (InertiaCube3) on upper limb	1H	Hand-to-mouth	Upper limb position	Motion capture	–
[67]	Kinect v1 + 1 x-IMU	1H	Knee flexion/extension	Knee angle	Motion capture	RMSE $<14.2^\circ$
[68]	Kinect v1 Vs Kinovea (MBS) Vs Hillcrest (IMU)	2H	Walking	Hip and knee angles	Gait rehabilitation	RMSE $=3^\circ$ (IMU) RMSE $=10^\circ$ (Kin.)

Participants are labeled as healthy (H), Parkinsons (PD), injured (INJ), in rehabilitation (REH) and amputees (AMP). All experiments were conducted in lab unless otherwise stated. Reported performance metrics include Root Mean Square Error (RMSE), Interclass Correlation Coefficient (ICC), Coefficient of Variation (CV), Limits of Agreement (LoA), Standard Deviation (SDev) and Pearson’s correlation coefficient (r)

### Video-based motion capture

The use of cameras and computer vision algorithms for the analysis of human motion is a well-established application field, and has notable contributions from both research and industry [17]. Video-based motion capture and Marker-Based Stereophotogrammetry systems (MBS) are now the de-facto standard for high-precision applications, including biomechanics research and clinical gait analysis [4].

In MBS systems, multiple cameras employ Infra-Red (IR) illuminators and triangulation algorithms to track the 3D position of reflective markers moving within a calibrated field of view. When used for human motion capture, the subject is instrumented with a set of reflective markers to identify and track relevant anatomical landmarks, and the system uses their positions to reconstruct and track subject’s body segments and joints [18]. These systems have been proven to offer accurate and reliable motion tracking and are being widely used in human motion research and clinical studies. The established accuracy is less than 1 mm error for the position of single markers, which translates in the errors in the range of 1–4 degrees for the estimation of joint angles, according to the specific marker cluster configuration [18, 19]. There are a number of commercially available systems, equipped with high-performance cameras and different software solutions for out of the box motion analysis, including Vicon Nexus (Vicon Motion Systems, UK), Elite (BTSengineering, Milan) and Optitrack Motive (NaturalPoint, USA).

The main downside of the MBS is the high cost and the complexity of its setup and use. To address these issues, research solutions explore the use of marker-less motion capture systems and their integration with depth sensors [20, 21]. Despite promising results, the accuracy and reliability implied with these new techniques do not yet meet the needs of healthcare applications, due to cumbersome hardware and extensive data processing requirements [22, 23].

### Low-cost video sensing: the Kinect

Microsoft first introduced the Kinect sensor in November 2010 to be used as a motion capture input device, as an add-on for the Xbox game console. It featured a standard digital video camera, a depth sensor based on structured IR illumination, and a directional microphone. The integration of the Kinect with dedicated algorithms allowed marker-less tracking of the user’s segments pose and movements, creating a natural user interface based on gestures [24]. Although it was developed and sold as a game controller, its offer of RGB video and IR-based depth sensing (RGB+D), at a very low price, made it appealing for a wide range of users, also in biomechanical and clinical research [25, 26]. With the availability of drivers and of a Software Development Kit (SDK) for a more general use beyond gaming, the Kinect has been applied to a vast range of academic and industrial projects, including the fields of interaction, robotics and, in fact, biomechanics [27]. The first version of the Kinect (Kinect v1) was followed by a re-designed sensor presented in 2013 (Kinect v2), which introduced an improved RGB camera and a new IR time-of-flight depth sensor [28]. Kinect v2 and its new SDK improved the sensor’s tracking capabilities and enhanced its use in applications based on human motion tracking [29].

The Kinect sensors have been extensively evaluated in relation to several application fields. The accuracy of the sensors and their depth estimation capabilities have been analyzed carefully [30], as well as the differences between the two versions [31–33]. Focusing on human motion capture applications, the use of the Kinect v1 in such scenario was triggered by the release of reverse-engineered open-source drivers and tracking software [34] and then propelled by the release of the Microsoft’s SDK [35]. The second-generation device and its updated algorithm have been validated further within the context of clinical motion analysis, with applications such as posture and balance evaluation [36, 37], fall detection [38], rehabilitation exercises [39–41], and gait assessment [42–44]. Moreover, the usability of Kinect-based home rehabilitation systems has been investigated, providing insights on the user acceptance with good results and indications for future improvements [45, 46].

The two generations of Kinect sensors have been compared in validation studies: when applied to posture or movement evaluations, these showed similar results, with the Kinect v2 just slightly outperforming its predecessor [47, 48]. The new sensor achieved good overall performance in the tracking of human pose and elementary movements, but showed obvious limits when dealing with more complex exercises or when the movements were not performed with the subject standing facing the sensor. These results necessarily reduce the use of the Kinect as an accurate tool for possible exploitations in the clinical context, but open the door for a possible use in somehow qualitative evaluations of posture and exercise, and also show the potential of such system for at-home monitoring of rehabilitation therapies.

### Inertial-based motion capture

The availability of Microelectromechanical Systems (MEMS) and their development for miniaturized sensors, combined with integrated processing and communication technologies, enabled the development of wearable sensing devices for human body monitoring [1, 15]. To obtain information regarding specific human locomotion parameters, one or more sensing devices are worn directly on relevant body parts and connected to a central processing hub for data collection and processing, forming a so-called Body Sensor Network [69]. However, this multi-sensor setup presents a number of technological requirements in terms of sensing capabilities, signal bandwidth, throughput and other general challenges such as device wearability, system usability, and data reliability [70].

There are several commercial examples, starting with high-end solutions for body motion capture [71], which are mainly used for animation and clinical movement analysis, all the way to ubiquitous motion trackers and sensors embedded in smartphones [72]. Notable examples include MVN Biomech (Xsens Technologies, Netherlands) and Opal (APDM Technologies, USA). The research and academic community is also very active on this topic, with several proposed platforms [73–78].

A wearable IMU provides unobtrusive methods to collect motion data relative to the body segment where it is worn; by combining a network of sensors, to form a whole-body model, joint motion can also be deduced. The integration of multiple sensors within the same device (accelerometer, gyroscope and magnetometer) allows to deploy robust sensor fusion algorithms in order to provide reliable and detailed information in a wide range of dynamic conditions and application contexts. In biomechanics, the most used application is the estimation of the device’s orientation from the embedded sensors and its use for the estimation of joint angles [79, 80]. Algorithms derived from navigation applications are adapted to infer the orientation of the body segment of interest and include the Kalman Filter (KF), its extended and unscented variations, and also several implementations of Complementary Filters (CF) [81–84]. Moreover, IMU sensor data can be exploited to analyze various features of human motion and dedicated algorithms have been developed for tasks such as activity recognition [85], exercise recognition and evaluation [86, 87], gait analysis [63, 88, 89] and jump analysis [90, 91].

Research and clinical studies have validated the use of wearable IMUs also in various conditions and applications [80, 92]. Notable examples include balance and postural evaluation [93, 94], fall monitoring and prediction [95], gait analysis [96] and rehabilitation [64]. Laboratory evaluations and comparisons with high-precision MBS systems have shown high accuracy and reliability of wearable motion sensors. Hence, these can be used in clinical practice for the evaluation of human motion and can provide a valuable and portable tool for standardized motor tests [97]. Usability aspects of the employment of such systems for home rehabilitation evaluation have also been investigated providing encouraging results [98–100]. However, the scientific community still faces the challenges implied in the development of accurate, reliable and easy to use wearable solutions for motion analysis, and in their extensive validation in real-life contexts.

Another emerging approach is to combine the outputs of the two systems [66, 67, 101]. In [67] the authors propose a sensor fusion algorithm to combine the Kinect and a set of wearable IMUs showing how the combined result achieves higher accuracy than any of the two systems. Additionally, in [66] an integration of IMUs and Kinect for the tracking of upper limb motions is proposed, showing again improved results when compared to the two separate systems. The work in [68] compares the use of IMUs and a Kinect-based system (Reha@Home) for gait analysis. This comparison, however, was limited to only one subject performing a short walk in front of the camera. In addition, the typical major problem of Kinect, i.e., the occlusions of body segments during the motion exercise, was promoted by the experimental setup adopted and the exercise performed.

Separately, the different systems have been extensively evaluated, but direct comparison and the discussion of their tradeoffs are still very limited. Moreover, all the reported studies focus on laboratory-based validation, despite huge potential of such systems lies in at-home use and therefore these systems should be evaluated also in this context. This work provides a detailed analysis of the literature on the two approaches (see Table 1) and an original comparison performed both in laboratory with a state-of-the-art motion tracking reference and in different home settings.

## Review of segment and joint kinematics estimation algorithms

The estimation and tracking of human segment and joint kinematics using video or wearable sensing is a well-documented research field, with several available solutions. This section will outline the main methods used in this work with the Kinect and with inertial sensors, whose estimates will be directly compared.

### Kinect

Microsoft provides a comprehensive SDK for the Kinect, which includes a ready-to-use algorithm for the estimation and tracking of the user’s complete body pose. The latest update provides real-time tracking for up to six people and it provides estimated 3D position for a complete skeletal model formed of 21 body joints and quaternion-based rotations of the relevant segments. The algorithm is based on the identification of the different body segments from the RGB+D video stream and uses a Random Forest recognition approach, which was trained with a wide dataset composed by real and synthetic data [24]. The research community has proposed some alternatives and there is still on-going work on pose estimation from RGB+D streams [102]. However, Microsoft’s solution is the de-facto standard thanks to its robustness and ease of use. For these reasons, it was used in several validation and exploitation studies [36, 39, 42, 43] and is also used in the present work. The provided data are only low-pass filtered to eliminate noise. Further offline smoothing or any other processing was avoided because, in the present study, real-time tracking of the exercise was targeted.

### Inertial sensing

Most of the previous validation studies used commercial solutions to obtain the orientation of the wearable sensors, which are used to estimate the orientation of the body segment they are attached to. Their outputs are then combined to form a partial or complete body pose estimation, based on the number of sensors in use [9, 59–64]. While there are several proposals for algorithms for the estimation of orientation from inertial sensors’ data, the present work analyzes the most used ones, to provide a comparative analysis targeting robust and well-established solutions. Moreover, to evaluate the standard use at-home of these systems, robust but ready-to-use approaches, without the need for system calibration or additional operations, were considered. In particular, although all the proposed methods provide the full orientation of the device, its horizontal component is not frequently considered, since it is heavily affected by the environmental ferro-magnetic disturbances. Although inertial and magnetic sensors may be influenced by the environment (e.g., temperature [103, 104]), environment-aware calibration and rejection techniques are out of the scope of the present work. The EXLs3 sensors used in the present study are calibrated in factory, and all the experiments had a limited duration with standard and stationary environmental conditions, therefore effects from the environment are assumed to be null.

All the orientation estimation algorithms are based on a combination of triaxial sensor inputs composed by accelerometer readings ${\varvec{a}} = \{a_x,a_y,a_z\}$, gyroscope readings ${\varvec{\omega }} = \{ \omega _x, \omega _y, \omega _z\}$ and magnetometer readings ${\varvec{m}} = \{m_x,m_y,m_z\}$, providing as output the orientation of the sensor, expressed either in quaternions (${\varvec{q}} = \{q_0,q_1,q_2,q_3\}$), Euler angles (${\varvec{E}} = \{E_x,E_y,E_z\}$) or rotation matrix (${\varvec{R}}$). Each of these three orientation representation methods has its advantages, but usually the quaternions are preferred for the computation efficiency and the results are converted to Euler angles because of their better clarity [105].

#### Orientation estimation from accelerometer (ACC)

Using accelerometer (or accelerometer and magnetometer) outputs, the sensor’s orientation is estimated by applying trigonometric functions. This approach assumes that the accelerometer is measuring only the gravity acceleration, and hence it is reliable only in static conditions. Accelerometer readings are used to estimate a partial orientation of the device, ${\varvec{E}}^a$ as

1 $$\begin{aligned} E^a_x\,=\, & {} atan2(a_y,a_z) \end{aligned}$$

2 $$\begin{aligned} E^a_y\,= \,& {} atan2(a_x, \sqrt{a_y^2 + a_z^2}). \end{aligned}$$

From the magnetometer measures, the missing horizontal heading is estimated as

3 $$\begin{aligned} E_z\,=\, & {} atan2(-m^a_y,m^a_x) \end{aligned}$$

where ${\varvec{m^a}}$ is the magnetometer reading projected to the accelerometer-estimated orientation plane identified by ${\varvec{E}}^a$.

#### Gyroscope integration (GYR)

Orientation of the sensor can also be estimated by integration of the angular velocity provided by the gyroscope. This estimate is reliable in dynamic situations, but suffers from drifts due to numerical integration errors. According to the chosen orientation representation, there are several implementations of its derivative; here, the quaternion one is adopted resulting in ${\varvec{{\dot{q}}}} = {\varvec{\Omega }}{\varvec{q}}$, which is integrated as

4 $$\begin{aligned} {\varvec{q}}(t)= & {} \left({\varvec{I}} + \frac{1}{2}{\varvec{\Omega }} dt\right) {\varvec{q}}(t-dt); \end{aligned}$$

5 $$\begin{aligned} {\varvec{\Omega }}= & {} \begin{bmatrix} 0 &{} -\omega _x &{} -\omega _y &{} -\omega _z \\ \omega _x &{} 0 &{} \omega _z &{} -\omega _y \\ \omega _y &{} -\omega _z &{} 0 &{} \omega _x \\ \omega _z &{} \omega _y &{} -\omega _x &{} 0 \\ \end{bmatrix} \end{aligned}$$

where ${\varvec{I}}$ is a $(4 \times 4)$ identity matrix and ${\varvec{q}}(0)$ computed using the ACC estimation during a short static initialization.

#### Kalman filter (KF)

The Kalman filter is a widely used approach for optimal fusion of accelerometer and gyroscope orientation estimates [79, 81]. Several variations have been proposed and here a straightforward application of a quaternion-based KF is applied, using the GYR derivate as the state equation, which is then corrected by the ACC measurement. The KF state and measurement equations are implemented as

6 $$\begin{aligned} {\left\{ \begin{array}{ll} {\varvec{{\dot{q}}}}(t) = {\varvec{\Omega }}{\varvec{q}}(t) + {\varvec{w}}(t) \\ {\varvec{q}}_a(t) = {\varvec{q}}(t) + {\varvec{v}}(t), \end{array}\right. } \end{aligned}$$

where ${\varvec{q}}(t)$ is the state estimate, ${\varvec{w}}\sim {\mathcal {N}}(0,{\varvec{R}})$ the zero-mean gaussian process noise with covariance matrix ${\varvec{R}}$, ${\varvec{q}}_a$ the accelerometer-based orientation estimate and ${\varvec{v}}\sim {\mathcal {N}}(0,{\varvec{Q}})$ the measurement noise with covariance matrix ${\varvec{Q}}$. The two covariance matrices were set to be diagonal with constant coefficients: 0.0001 for Q and 0.1 for R.

#### Madgwick filter (MAD)

Another approach for a quaternion-based iterative fusion of ACC and GYR estimates has been proposed by Madgwick [84] and it has been well received because of its high-quality estimate and limited computational and memory requirements. It is based on a gradient descent algorithm, which iteratively finds the optimal orientation given the input signals and it is governed by the following differential equations:

7 $$\begin{aligned} {\varvec{q}}(t)\,=\, & {} {\varvec{q}}(t-1) + {\varvec{{\dot{q}}}}_{est}(t) dt \end{aligned}$$

8 $$\begin{aligned} {\varvec{{\dot{q}}}}_{est}(t)\,=\, & {} {\varvec{{\dot{q}}}}_{\omega }(t) -\beta {\varvec{{\dot{q}}}}_{a}(t) \end{aligned}$$

9 $$\begin{aligned} {\varvec{{\dot{q}}}}_{a}(t)\,=\, & {} \frac{\nabla f}{\Vert \nabla f\Vert }. \end{aligned}$$

The filter calculates the orientation ${\varvec{q}}$ by numerically integrating the estimated orientation rate ${\varvec{{\dot{q}}}}_{est}$, which is computed as the rate of change of orientation measured by the gyroscopes, ${\varvec{{\dot{q}}}}_{\omega }$, with the magnitude of the gyroscope measurement error, $\beta$, removed in the direction of the estimated error, ${\varvec{{\dot{q}}}}_{a}$, computed from accelerometer and magnetometer measurements. ${\varvec{{\dot{q}}}}_{a}$ is computed with the gradient descent method and *f* represents the function that provides the orientation from accelerometer and magnetometer readings. The implementation of the algorithm makes use of established matrix and quaternion operations, and the correction parameter $\beta$ was empirically set to 0.01 [84].

#### Complementary filter (CF)

Another class of orientation estimation algorithms was developed by Mahony et al. using non-linear complementary filters [83]. Such approach is also becoming popular for its accuracy and reduced computational complexity when compared to KF. In this case, a rotation matrix representation is used and, contrary to the KF, the filter combines accelerometer and gyroscope estimates with a constant correction factor, following the equation:

10 $$\begin{aligned} {\varvec{{\dot{R}}}}\,=\, & {} [{\varvec{R}} [\omega ]_{\text {x}} + k_p {\varvec{R}} \gamma ]_{\text {x}} {\varvec{R}}; \end{aligned}$$

11 $$\begin{aligned} \quad [{\varvec{\omega }}]_{\text {x}}= & {} \begin{bmatrix} 0 &{} -\omega _z &{} \omega _y \\ \omega _z &{} 0 &{} -\omega _x \\ -\omega _y &{} \omega _x &{} 0 \end{bmatrix} \end{aligned}$$

where ${\varvec{R}}$ is the rotation matrix, $k_p$ the filter gain empirically set to 0.5 and $\gamma$ the correction term given by the difference of the previous estimate and the current one from the accelerometer [83].

## Results

The experimental part of this work directly compared the two systems in a laboratory setting, using a high-precision MBS tracking system together with an internationally established technique as the gold standard. In addition, the use of these two systems out of the laboratory was compared, performing a pilot evaluation in unconstrained environments such as patient’s house. Detailed description of experimental methodology and protocols is provided in “Methods” section.

### Laboratory evaluation

Table 2 reports the mean differences, i.e., root mean square errors (RMSE), and the standard deviation for the considered techniques for IMU orientation estimation and for the estimates provided by the Kinect, when compared to the corresponding results established through MBS. The different IMU approaches have a similar performance, with the KF and MAD algorithms outperforming the others. It is interesting to note that the single-sensor algorithms have only limited degradation compared to the sensor fusion ones and in some cases even outperform the CF. This is mainly due to a combination of the type of performed exercises (large motions with a relatively low dynamic) and their short duration, which allow also ACC or GYR estimates to have limited RMSE. The IMU estimates employing sensor fusion algorithms outperform the Kinect’s output, though by a limited margin, revealing that both approaches (by sensors or cameras) have a good overall performance, with errors in the range of 3 to 8 degree for all the joint angles analyzed, which is consistent with existing literature [39, 55, 59–61, 68].

**Table 2:** Mean errors for various IMU orientation estimation algorithms and for the Kinect v2 when compared to the MBS output

	Trunk Sag.		Trunk Front.		Hip Sag.		Hip Front.		Knee Flex.		Mean	
	GA	GAC	GA	GAC	GA	GAC	GA	GAC	GA	GAC	GA	GAC
ACC	3.3 (1.5)	5.1 (1.9)	5.9 (3.2)	7.0 (2.5)	9.3 (4.2)	8.1 (2.2)	8.8 (2.8)	11.4 (5.3)	9.4 (3.5)	12.3 (2.3)	7.4 (3.0)	8.8 (2.8)
GYR	5.2 (2.8)	5.1 (1.3)	6.3 (2.1)	5.9 (2.3)	9.7 (3.6)	8.7 (2.7)	8.3 (2.8)	11.1 (5.3)	7.4 (2.9)	8.6 (4.4)	7.4 (2.8)	7.7 (3.2)
KF	2.7 (1.0)	4.4 (1.8)	5.2 (3.2)	6.5 (2.4)	7.8 (3.4)	6.2 (1.3)	8.1 (2.8)	10.5 (5.9)	5.1 (2.0)	8.3 (3.4)	5.9 (2.5)	7.2 (2.9)
MAD	2.4 (0.9)	4.1 (1.5)	4.6 (3.0)	5.9 (2.2)	8.0 (3.3)	6.6 (1.1)	8.0 (2.9)	10.2 (6.3)	5.3 (2.3)	8.0 (4.6)	5.5 (2.3)	6.9 (3.1)
CF	3.4 (1.5)	5.1 (1.9)	6.3 (3.3)	7.1 (2.5)	9.5 (3.8)	8.5 (2.1)	9.5 (2.8)	11.6 (5.3)	10.5 (3.9)	12.7 (2.5)	7.9 (3.1)	9.00 (2.9)
Kinect	2.9 (0.9)	2.7 (1.2)	3.7 (1.3)	3.8 (1.1)	7.0 (3.2)	5.8 (3.4)	8.6 (3.4)	10.3 (6.0)	5.7 (1.4)	7.1 (2.1)	5.6 (2.0)	5.7 (2.8)
Mean	3.3 (1.4)	4.4 (1.6)	5.3 (2.7)	6.0 (2.1)	8.6 (3.6)	7.3 (2.1)	8.6 (2.9)	10.7 (5.7)	7.3 (2.7)	9.3 (3.2)	6.6 (2.7)	7.6 (3.0)

The standard gait analysis while undressed (GA) and to the one with clothing (GAC) is reported. Unit is degree, mean (and std) over all the performed exercises are reported

**Fig. 1:**
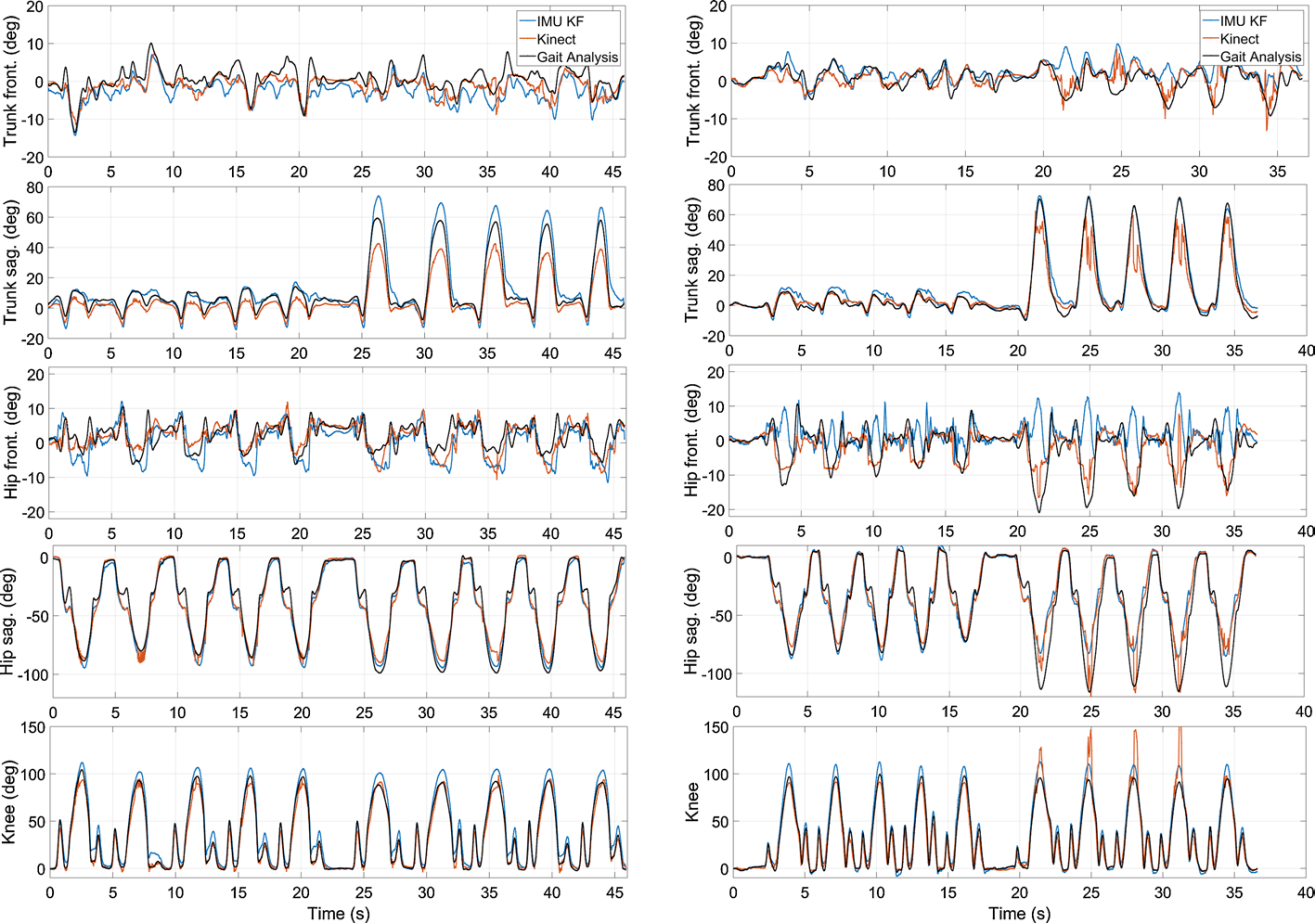
Sample data for a lunge exercise as performed in the lab without clothes (left), and with clothes (right), as monitored by wearable IMUs (IMU KF), Kinect v2 and gait analysis. The plots show in series over time 5 repetitions of normal execution, followed by 5 repetitions with larger forward inclination of the trunk

As expected, the exercises performed while wearing clothes show a slightly higher RMSE and deviations; however, they are consistent with the standard GA case. The comparison between the different approaches confirms again that the sensors fusion algorithms outperform the single-sensor estimates and are aligned with the Kinect ones. Given the limited sample size, a thorough analysis on the performance degradation cannot be provided; however, additional errors in this case can be attributed to the motion artifacts caused by clothing, which allows for relative motion also between the markers and the underlying anatomical landmarks and also between IMUs and markers. Figure 1 shows resulting angles for the frontal lunge exercise in standard gait analysis while undressed (left), and the corresponding with clothing (right) from the same subject. Patterns from both IMU and Kinect match generally well with the gold standard from gait analysis, with consistent results for all the executed exercises and across all users. Timing of the waveforms is exactly the same, whereas peak values show differences, though consistent over repetitions. These can be accounted for the different technique applied for calculation of orientation, i.e., IMU tracking a single limited area of the body segment, while the Kinect is searching for the overall orientation of this segment, this being affected also by its deformation during motion.

### At-Home evaluation

**Fig. 2:**
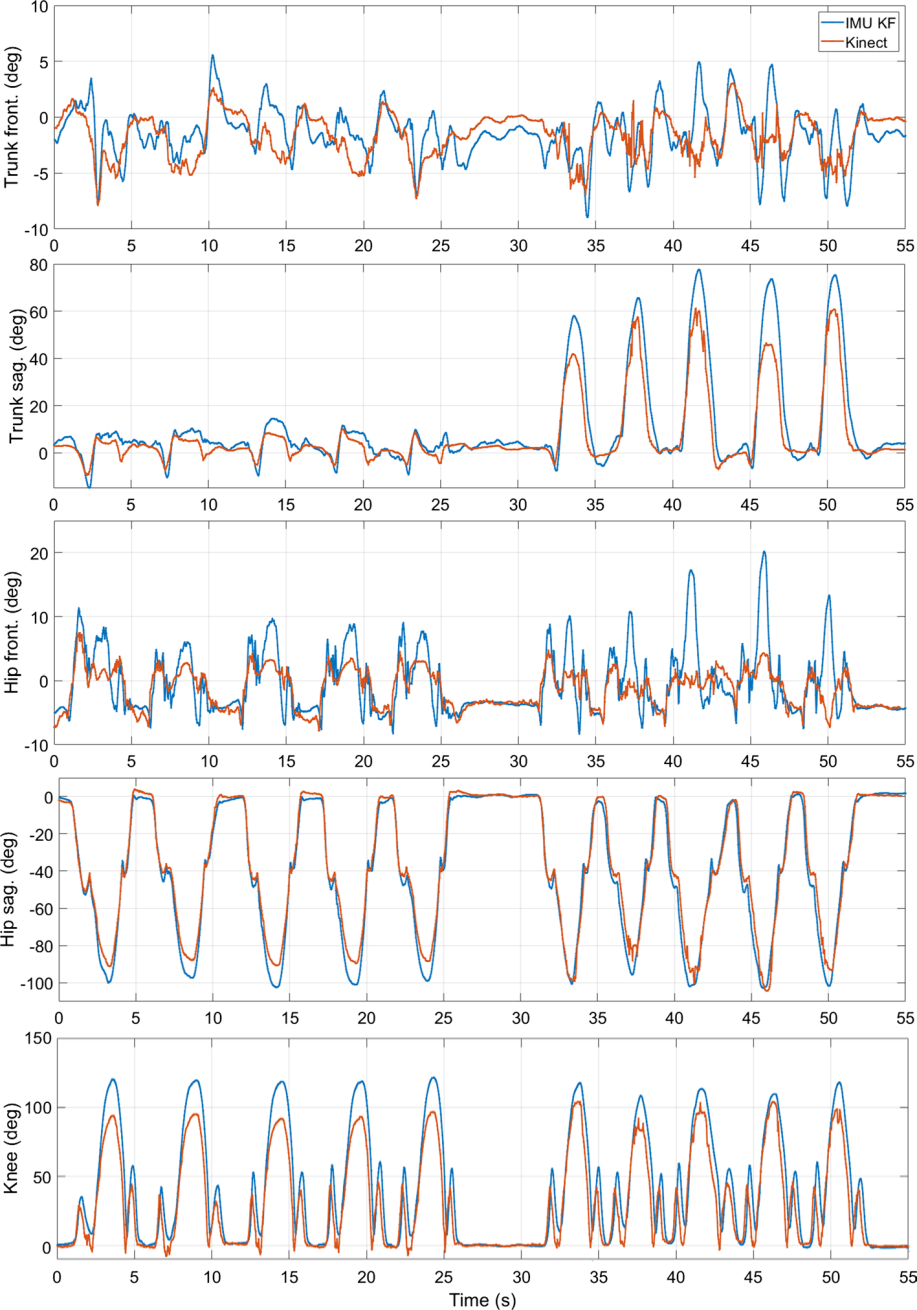
Sample data for a lunge exercise as performed at home and monitored by wearable IMUs (IMU KF) and the Kinect v2. The plots show in series over time 5 repetitions of normal execution, followed by 5 repetitions with larger forward inclination of the trunk

Without the availability of a gold standard, it is not possible to calculate errors for the two systems for a quantitative evaluation. However, it is possible to qualitatively evaluate the outcomes and compare them to the data collected in the lab. In particular, it is possible to compute the average difference and standard deviation between the two estimates and use such parameters to compare the lab and home sessions. Table 3 collects the root mean square difference, its standard deviation and the maximum differences between IMU and Kinect estimates. For the three subjects who performed the exercises both in the lab and at home, there is a direct comparison of the two environments, while the average results for all sessions performed at home provide a qualitative insight of the performance outside the lab. A sample of the joint angles resulting from at-home acquisitions is plotted in Fig. 2, where the frontal lunge exercise is shown to facilitate the comparison with Fig. 1 showing the same exercise from the same subject performed in the lab.

**Table 3:** Differences between the IMU KF and Kinect estimates during lab and at-home tests for each of the three subjects analyzed

	Trunk Sag.			Trunk Front.			Hip Sag.			Hip Front.			Knee Flex.			Mean		
	RMSD	Std.	Max	RMSD	Std.	Max	RMSD	Std.	Max	RMSD	Std.	Max	RMSD	Std.	Max	RMSD	Std.	Max
S1																		
Lab	2.3	0.6	14.5	6.4	4.7	26.9	5.3	2.1	49.2	4.9	0.8	46.1	7.3	1.2	75.5	5.3	1.9	42.4
Home	2.0	1.2	11.2	5.2	3.0	27.3	8.3	4.6	41.7	13.6	6.1	56.3	15.8	7.5	81.7	9.0	4.5	43.6
S2																		
Lab	2.6	0.8	15.8	4.4	2.6	25.9	5.9	1.7	29.2	5.9	0.7	35.7	8.3	1.5	72.0	5.4	1.5	35.7
Home	1.9	0.7	6.5	5.3	3.6	13.8	12.5	4.4	41.8	8.8	4.3	30.0	14.1	4.3	47.5	8.5	3.5	27.9
S3																		
Lab	3.2	0.9	12.1	4.6	1.3	14.2	8.8	4.8	29.3	6.7	1.7	18.7	5.3	1.6	24.6	5.7	2.1	19.8
Home	2.3	0.4	8.5	3.9	0.6	9.4	5.5	2.3	20.4	5.9	4.0	21.6	15.1	4.8	40.7	6.5	2.4	20.1
All																		
Lab	2.9	0.9	14.4	5.3	2.7	22.1	6.4	2.7	33.9	5.7	1.2	31.5	7.1	1.7	54.7	5.5	1.8	31.3
Home	2.1	0.7	8.7	4.8	2.4	16.8	8.8	3.8	34.6	9.4	4.8	36.0	15.0	5.5	56.6	8.0	3.5	30.6

Root Mean Square Difference (RMSD), standard deviation and maximum difference between the two systems are reported. Unit is degree

All the performed exercises were correctly acquired by both systems in all the four test environments. A comparison of the outputs shows that they reported expected outcomes and the two systems show again similar performance. For the subjects monitored both in lab and at home, the difference between the two systems is consistent in both cases, with the exception of the knee flexion angle, which exhibits higher deviations in the un-controlled environments. The same outcomes are observed also for the average over all the subjects who performed exercises in the home environments. Considering both Figs. 1 and 2, it emerges that the systems show a difference in the estimated range of motion, with the Kinect underestimating it when compared to both the MBS reference and the IMU output. Similar results were observed in all acquired sessions, though such behavior should be analyzed further to establish systematic evaluations of the outcomes from the two systems. Moreover, Kinect-based estimates show also considerable peak discontinuities, as depicted in Fig. 2, right column. This result was observed throughout the dataset and it can be caused by image glitches disturbing the vision-based tracking algorithm. Of course, this influences the reported measurements of estimate differences. To mitigate this effect, ad-hoc filtering or smoothing techniques may be applied in the future.

## Discussion

In the last decade, the consumer market opened the way for a broader acceptance and use of wearable sensing devices. Activity trackers are now widely employed in everyday life, but with limited reliability and validation of results [11, 106]. More accurate wearable inertial sensors have been adopted for a wide range of clinical applications [2, 107], with a huge potential to innovate and improve nearly every aspect of healthcare applications. But for a successful exploitation of these systems in healthcare and in particular in rehabilitation, there is definitely the need for their careful quantitative validation. In addition to these, unobtrusive sensing systems based on video and depth cameras are available at a low price and high performance, such as the Kinect v2 here assessed. It was originally developed as an interaction controller for home video games, but it has gained attention also for general research and clinical applications for its capability to track human subjects’ movements in real time [27, 108]. With respect to inertial sensors, video-based tracking is even less invasive, as the body of the tracked subject is free of any instrument.

Several studies have analyzed the performance and validated these two systems for the tracking of human motion in clinical applications, including postural and balance control, rehabilitation exercises, gait, or specific conditions such as Parkinson’s or post stroke rehabilitation. Laboratory tests showed the limits of the low-cost tracking technologies when compared to state-of-the-art MBS systems; however, these also highlighted their overall applicability to ubiquitous patient monitoring (see Table 1 and the references therein). The development and adoption of innovative monitoring systems for effective patient monitoring in unconstrained environments open new research challenges in these systems’ reliability, sensitivity to environmental and operational factors, usability and acceptability by the clinicians and the end users, i.e., the patients.

In the present study, a thorough experimental analysis was performed to assess the accuracy of two instruments for human motion tracking in the context of rehabilitation. The experiments are established however as preliminary measurements on a limited sample size. Nevertheless, the state-of-the-art gait analysis was arranged as gold standard, and a large number of exercises were analyzed. These were a limited specific set within all possible rehabilitation exercises, particularly used to recover from a large number of orthopedic disorders and treatments. The scope in fact was to test the two instruments in a number of general yet well representative motor tasks; in the future these two instruments shall be tested also in other possible exercises. It is important to note, however, that among those analyzed here, the squat position is definitely very physically demanding for the extreme joint positions implied, and as such particularly suitable to reveal large measurement differences. As additional limitation, the two clothing conditions were tested in a single subject only, but this was thought just to reveal the additional artifact introduced by the clothes used routinely in these exercises, knowing that the gold standard for these measurements is represented by the motion at the skeletal system.

Validation against state-of-the-art gait analysis was performed in two different conditions, though in a very small number of subjects. The standard procedure always requires the subject to be undressed, with all the markers attached to the skin in correspondence of relevant anatomical landmarks. This is recommended for a repeatable application of the marker set (for intra- and inter-subject comparisons) and to avoid the disturbances of the clothing, which adds considerable artifactual measurements. However, this is not the typical condition for the users of these systems; therefore, the validation was repeated, in one subject, also imitating a more realistic dressing condition, with the user wearing comfortable fitness clothing, typical of physical exercises in the gym or at home. In the latter case, the measures were less accurate, but they are more representative of a real scenario. The preliminary results here reported for the two systems highlight the importance of instructing the users to perform the exercises with limited and appropriate clothing and to tightly wear the sensors to limit occlusions and motion artifacts. Although all the present sensing technologies are likely to be affected by environmental factors (e.g., temperature, humidity, etc.) and by their status (duration of use, etc.), a detailed analysis of such influences is out of the scopes of this work. The present experimental protocol was rather designed to minimize the impact of any such external factors and environmental conditions. Moreover, the aim was to limit the differences between the acquired sessions and with respect to the corresponding conditions in the relevant literature (Table 1).

The two systems showed similar performance in terms of final angle estimations when considering simple large-motion exercises. The measurements from this experimental work on both the laboratory and at-home sessions show good repeatability and consistency, therefore providing reliable evaluation of the performance of relevant rehabilitation exercises. However, the results also showed differences in the body segment orientations and therefore joint rotations, but these are consistent and small with respect to the corresponding overall range of motion. These findings are aligned with the reported literature, which generally reports errors below 10 degrees [40, 109].

Today, there is no consensus on the necessary accuracy that these motion tracking systems should provide for these to be appropriate in physical rehabilitation. However, based on the existing literature [23, 110], reports from therapists and physicians, as well as practical experience, errors in human segment or joint rotations smaller than 3 degrees would be tolerable for most rehabilitation programs in orthopedics; errors between 3 and 6 degrees can still be acceptable, depending on the joint, the pathology and treatment, and the status of the patient. For example, after the replacement of shoulder, hip and knee joints, the range of motion usually restored is far larger than 100 degrees, and this error therefore would be only a very small percentage. In this context, the two analyzed technologies perform well, and the errors here revealed can be well acceptable in most major human diarthrodial joints, compatible with the status of the patient and the rehabilitation exercises under observation. Direct or indirect, i.e., for at-home sessions, careful supervision and evaluation should be guaranteed in any case by trained therapists. This is in any case a step forward with respect to qualitative observations, which is biased by therapist experience.

Nevertheless, the different basic technology of these two systems introduces additional considerations on their effective use. The Kinect is a well-supported commercial platform and benefits from its very simple operational requirements. To track movements, it just needs to be placed at 3–4 m in front of the subject and connected to a personal computer, without the need for additional instrumentation or further requirements. However, its vision-based approach imposes a limit on the tracked area, particularly a frontal view, and no object interposition; also, its low sampling frequency limits the range of movements correctly tracked. In particular, fast and complex movements as well as those with large components out-of-the-frontal plane of the sensor are not tracked by the system [44], thus precluding its use in applications such as real-life monitoring of patients and rehabilitation exercises performed while lying or with support devices. In addition, its limited field of view precludes its use for unconstrained gait monitoring.

Wearable IMUs are now a mature and widely adopted technology, with several commercial solutions ranging from whole-body motion tracking suites to sensor kits and stand-alone units. The use of IMUs attached to a target body segment and the adoption of relevant sensor fusion algorithms is nowadays commonly employed to analyze human motion within a large spectrum of motor tasks and exercises, from up-right posture to complex sports activities [109, 111, 112]. IMU use for clinical motion analysis has been extensively evaluated regarding accuracy and reliability, but evaluation studies are mostly confined to laboratories [64, 93, 96]. Considering at-home uses, wearable IMUs have an additional requirement when compared to the Kinect, since the user has to wear the sensors. Such operation usually consists in mounting a simple elastic band, which can be considered simple enough for autonomous use at home even for children and elderly, but it can be, in theory, a source of uncertainty (i.e., sensor misplacement) or it can be problematic for severely impaired users. On the other hand, wearing the sensors on the user’s body allows for a less-constrained tracking and for the development of a mobile solution capable of acquiring movements in a truly unconstrained and pervasive manner. The vast range of available sensors, paired with state-of-the-art processing algorithms, allows for the development of diversified solutions covering a wide spectrum of human motions, including static and postural analysis, rehabilitation exercises, jump analysis, gait analysis, fall detection, etc.

## Conclusions

This work addresses two of the most promising technologies for at-home rehabilitation monitoring based on real-time motion analysis, i.e., wearable IMUs and Kinect. The Kinect incorporates video and depth sensors and provides easy to use, real-time, full-body tracking at a low price. Wearable inertial sensors are now emerging as another reliable tool for movement analysis, providing an additional instrument for patient monitoring also in clinical and research settings. In the first part of this study, a detailed critical analysis of the literature on these technologies was performed (see Table 1), and in the second part original comparisons between the two are reported, after thorough experiments performed both in a state-of-the-art motion capture laboratory and in direct home settings.

From the literature it emerged that the two different technologies have been assessed extensively, though mostly separately, with very limited direct experimental comparisons. In addition, only a few studies have addressed the final real conditions of use, i.e., at-home. Therefore, an original experimental analysis was performed, in both environments. The two systems showed similar performance in tracking elementary exercises with large range of motion, and provided comparable results both in the laboratory setting and in-home tests. In the former, IMUs combined with different sensor fusion algorithms showed an average RMSE of $5.5^\circ (\pm 2.3)$ over the performed exercises, which matches well with those from the Kinect, $5.6^\circ (\pm 2.0)$. These exercises were replicated with the same experimental protocol and with the same users in home environments, showing results much in support of those obtained in the laboratory.

The Kinect has the advantage of very simple operational requirements, but it lacks the capabilities to track complex and highly dynamic movements, especially when the user does not move in front of the sensor. On the other hand, IMUs must be worn, but work well in a large variety of human movements, also at high speed. Both technologies, however, can be adopted for home-based rehabilitation monitoring, after taking adequate precautions about user instructions and about correct interpretation of the results. With further developments and large-scale real-life evaluations, these technologies will allow careful and pervasive patient monitoring and relevant clinical studies in the near future.

## Methods

This section describes the methodology and the comparison protocols employed for the experimental analysis. Our institution’s Review Board (Comitato Etico dell’Istituto Ortopedico Rizzoli) approved the study conducted in the present work. All participants received detailed information about the study and provided written consent for the use of acquired data. All acquired data were anonymous and only age, gender, weight and height were stored along with the exercise data here reported. The subjects were recruited among graduate students at our institution.

### Laboratory evaluation

**Fig. 3:**
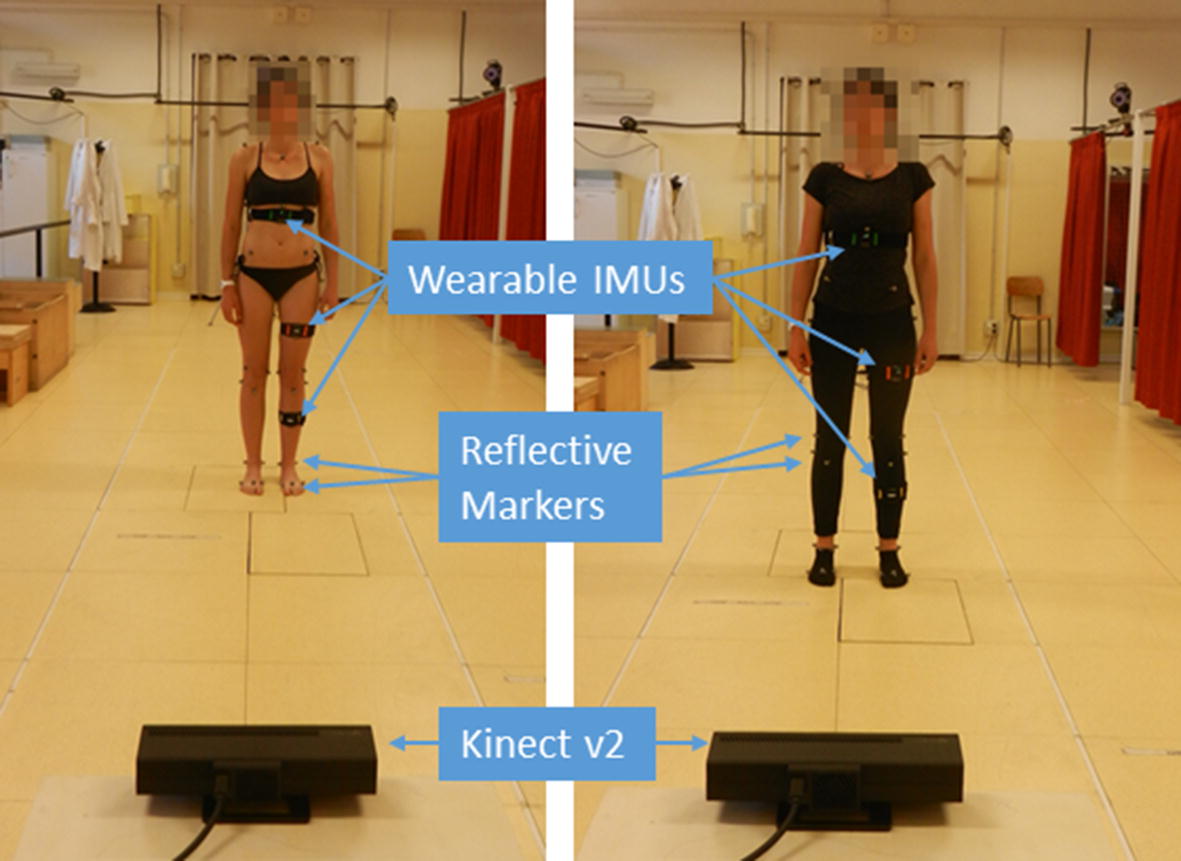
Data collection sessions in the gait analysis laboratory: the same overall setup with the instrumentation mounted on a subject for a standard gait analysis (left, undressed) and for more realistic final user condition (right, dressed). Instrumentation includes IMUs on relevant body segments and reflective markers on relevant anatomical landmarks according to the gait analysis protocol [113]

The direct instrumental comparison of the two systems was performed at the Movement Analysis Laboratory of the Rizzoli Orthopaedic Institute (Bologna, Italy) as shown in Fig. 3. Subjects’ motion was concurrently monitored by a Kinect v2 (Microsoft, Seattle, USA), a set of three EXLs3 wearable IMUs (Exel srl, Bologna, Italy) and a high-precision 8-camera MBS motion tracking system (Vicon 612, Vicon Motion Systems Ltd, Oxford, UK) sampling at 100 Hz.

During the acquisitions, the Kinect was placed in front of the subject, at a distance of approximately 3.50 m, and at 1 m from the ground (Fig. 3). It was checked whether the subject was at the center of the field of view of the sensor, as recommended from the product guidelines. The Kinect4Windows 2.0 SDK was used for data acquisition and processing. It provides the reconstruction of the full body segments, formed by the position and angles of 21 joints [24]. These data were saved for offline analysis by means of a custom application. The SDK does not allow control over data acquisition and it provides an approximate sampling rate of 30 Hz.

For IMU tracking, a 3-sensor kit of EXLs3 wireless IMUs was used. This study focused on the evaluation of lower limbs movements, hence the three sensors were placed on the frontal aspects of the subject’s thorax and of left thigh and shank. The devices are self-worn using elastic bands with a dedicated pocket for the IMU. Each EXLs3 device is calibrated in factory and provides an on-board estimation of its orientation, in addition to triaxial sensor data for accelerometer ($\pm 2~{\text{g}}$ full scale), gyroscope ($\pm 500~{\text{dps}}$ full scale) and magnetometer ($\pm 1200~\mu T$ full scale). These are equipped with a Bluetooth transceiver for data streaming to a host device. In the performed tests, sensor data were sampled at 100 Hz and streamed to a personal computer for offline analysis. Given the placement of the IMUs and combining the orientation of the three sensors, it is possible to estimate the thorax sagittal and frontal orientation, the hip joint sagittal and frontal angles and the knee joint flexion/extension.

As a gold standard reference, a state-of-the-art MBS motion capture system and an established gait analysis protocol were used. Before starting the data collection, 33 spherical 15-mm reflective markers were located on the lower limbs, pelvis and thorax in correspondence of known anatomical landmarks according to a validated protocol [113]. From these markers, anatomical-based reference frames were defined for each segment, and three-dimensional joint rotation angles were calculated according to international recommendations and conventions [114]. Thorax sagittal and frontal plane inclinations, hip joint sagittal and frontal angles and knee sagittal angle, i.e. flexion/extension, from these measurements and calculations were used as the gold standard for the comparison of the corresponding Kinect and IMU-based estimates. These gait analysis results were stored for offline comparative analysis.

The study involved three healthy subjects (female 1.75 m 26 years, female 1.65 m 31 years, male 1.83 m 34 years) who performed physical exercises typical of rehabilitation programs after replacement of lower limb joints. For all three, standard gait analysis was performed which implies instrumenting the subjects without clothing (Fig. 3 left). This is considered the optimal experimental setting, with the best possible accuracy of the measurements because of the direct attachment of the markers to the skin without interposition. For one of the three subjects gait analysis was repeated days later while wearing comfortable fitness clothing (Fig. 3 right). It is worth noting however, that when collecting data while wearing clothes, MBS measurements are likely to be affected by noise, since the markers are attached to the clothing and some tissue motion artifacts are inevitable. The subjects were wearing adherent fitness clothing, which can limit this motion artifacts.

The three subjects were first instructed about the functioning of the acquisition systems and how to wear the inertial sensors. In addition to squat (SQ), the following six exercises were performed by the left leg only: frontal lunge (FL), lateral lunge (LL), hip abduction (HA), hip flexion (HF), and hip extension (HE). These motion exercises include both basic and more complex movements and are typical of many rehabilitation programs targeting lower limbs functional recovery [115, 116]. For each exercise, the subjects were instructed to perform five repetitions as for standard correct execution first, i.e., with the trunk up-right, and then five more repetitions with the trunk in a large inclination forward, to mimic a common mistake in performing these rehabilitation exercises [115, 116]. The overall quality of the exercises was assessed by analyzing thorax orientation and hip and knee joint rotations; among these measurements, target parameters, i.e., those to determine the biofeedback, and control parameters, i.e., those to be checked for a correct performance of the exercise, are specified in Table 4.

**Table 4:** Collected exercises and corresponding target and control parameters

Exercise	Repetitions	Target param.	Control param.
Squat (SQ)	5 correct	Knee joint flexion	Trunk orientation
	5 forward thorax inclination,		
Frontal lunge (FL)	5 correct,	Knee joint flexion	Trunk orientation
	5 forward thorax inclination,		
Lateral lunge (LL)	5 correct,	Knee joint flexion	Trunk orientation
	5 forward thorax inclination,		
Hip abduction (HA)	5 correct,	Hip joint abduction	Trunk orientation,
	5 lateral thorax inclination,		knee joint flexion
	5 knee flexion		
Hip flexion (HF)	5 correct,	Hip joint flexion	Trunk orientation,
	5 backward thorax inclination,		knee joint flexion
	5 knee flexion		
Hip extension (HE)	5 correct,	Hip joint extension	Trunk orientation,
	5 forward thorax inclination,		knee joint flexion
	5 knee flexion		

Spatial and temporal alignment of the reference frames from the three systems was performed offline. A short static up-right double-leg posture of the subject was acquired at the beginning of each data collection session and used to align the body segment orientations provided by the three systems. Moreover, a sharp right leg movement was performed at the beginning of a session to facilitate offline time alignment of the data streams. All data were stored for offline analysis, which were performed in Matlab. For a direct comparison, the joint rotations streams from the three systems were all re-sampled at 30 Hz.

### At-Home evaluation

One of the main advantages of these two innovative approaches for human motion tracking is their low cost, which together with their small dimensions offer the possibility for ubiquitous adoption in rehabilitation centers, gyms and even at home. In addition to lab comparison, therefore, a pilot study was conducted to evaluate their use in the latter uncontrolled environment. To test the variability associated to different environmental conditions in real-life scenarios, the two systems were used to collect data in five additional locations. In particular, two homes and three different office spaces were used, where a total of 10 subjects were asked to perform the same set of exercises as during the laboratory evaluation. The same three subjects who performed the exercises in the laboratory were also among the home test group, to allow for a direct comparison of their performance. The spaces were different in dimensions and lighting conditions, going from a small office with artificial light to a large living room under direct sunlight. All sessions followed the same protocol as for the lab evaluation, except for the MBS-based gait analysis and the reference tracking, which was not available outside of the lab. At each location, the Kinect was positioned in the best position according to the environment and each user was asked to autonomously set up the IMU sensors while wearing comfortable fitness clothing and then asked to perform the exercises according to precise instructions by the operator.

## Data Availability

The datasets used and/or analyzed in the current study are available from the corresponding author on reasonable request.

## Abbreviations

IMU: Inertial measurement unit
MBS: Marker-based stereophotogrammetry
IR: Infra-red
RGB+D: RGB and depth
SDK: Software Development Kit
MEMS: Microelectromechanical system
KF: Kalman filter
CF: Complementary filter
ACC: Accelerometer
GYR: Gyroscope
MAD: Madgwick filter
SQ: Squat
FL: Frontal lunge
LL: Lateral lunge
HA: Hip abduction
HF: Hip flexion
HE: Hip extension

## References

[1] McGrath MJ, Scanaill CN. Wellness, fitness, and lifestyle sensing applications. In: Sensor technologies. Springer; 2013. p. 217–48.

[2] Zheng YL, Ding XR, Poon CCY, Lo BPL, Zhang H, Zhou XL (2014). Unobtrusive sensing and wearable devices for health informatics. IEEE Trans Biomed Eng.

[3] De Vito L, Postolache O, Rapuano S (2014). Measurements and sensors for motion tracking in motor rehabilitation. IEEE Instrum Meas Mag.

[4] Cappozzo A, Della Croce U, Leardini A, Chiari L (2005). Human movement analysis using stereophotogrammetry: part 1: theoretical background. Gait Posture.

[5] Moeslund TB, Hilton A, Krüger V (2006). A survey of advances in vision-based human motion capture and analysis. Comput Vis Image Underst.

[6] Saber-Sheikh K, Bryant EC, Glazzard C, Hamel A, Lee RY (2010). Feasibility of using inertial sensors to assess human movement. Manual Ther.

[7] Shull PB, Jirattigalachote W, Hunt MA, Cutkosky MR, Delp SL (2014). Quantified self and human movement: a review on the clinical impact of wearable sensing and feedback for gait analysis and intervention. Gait Posture.

[8] Leardini A, Lullini G, Giannini S, Berti L, Ortolani M, Caravaggi P (2014). Validation of the angular measurements of a new inertial-measurement-unit based rehabilitation system: comparison with state-of-the-art gait analysis. J Neuroeng Rehabil.

[9] Robert-Lachaine X, Mecheri H, Larue C, Plamondon A (2016). Validation of inertial measurement units with an optoelectronic system for whole-body motion analysis. Med Biol Eng Comput.

[10] Swan M (2012). Sensor mania! the internet of things, wearable computing, objective metrics, and the quantified self 2.0. J Sens Actuator Netw.

[11] Ferguson T, Rowlands AV, Olds T, Maher C (2015). The validity of consumer-level, activity monitors in healthy adults worn in free-living conditions: a cross-sectional study. Int J Behav Nutr Phys Act.

[12] Orwat C, Rashid A, Holtmann C, Wolk M, Scheermesser M, Kosow H (2010). Adopting pervasive computing for routine use in healthcare. IEEE Pervasive Comput.

[13] Banaee H, Ahmed MU, Loutfi A (2013). Data mining for wearable sensors in health monitoring systems: a review of recent trends and challenges. Sensors.

[14] Lange B, Chang CY, Suma E, Newman B, Rizzo AS, Bolas M. Development and evaluation of low cost game-based balance rehabilitation tool using the Microsoft Kinect sensor. In: Intl. Conf. of the IEEE engineering in medicine and biology society (EMBC). IEEE; 2011. p. 1831–4. 10.1109/IEMBS.2011.6090521 22254685

[15] Pantelopoulos A, Bourbakis NG (2010). A survey on wearable sensor-based systems for health monitoring and prognosis. IEEE Trans Syst Man Cybern Part C Appl Rev.

[16] Trombetta M, Henrique PPB, Brum MR, Colussi EL, De Marchi ACB, Rieder R (2017). Motion Rehab AVE 3D: a VR-based exergame for post-stroke rehabilitation. Comput Methods Prog Biomed.

[17] Poppe R (2007). Vision-based human motion analysis: an overview. Comput Vis Image Underst.

[18] Della Croce U, Leardini A, Chiari L, Cappozzo A (2005). Human movement analysis using stereophotogrammetry: part 4: assessment of anatomical landmark misplacement and its effects on joint kinematics. Gait Posture.

[19] Merriaux P, Dupuis Y, Boutteau R, Vasseur P, Savatier X (2017). A study of vicon system positioning performance. Sensors.

[20] Mündermann L, Corazza S, Andriacchi TP (2006). The evolution of methods for the capture of human movement leading to markerless motion capture for biomechanical applications. J Neuroeng Rehabil.

[21] Chen L, Wei H, Ferryman J (2013). A survey of human motion analysis using depth imagery. Pattern Recogn Lett.

[22] Corazza S, Muendermann L, Chaudhari A, Demattio T, Cobelli C, Andriacchi TP (2006). A markerless motion capture system to study musculoskeletal biomechanics: visual hull and simulated annealing approach. Ann Biomed Eng.

[23] Schmitz A, Ye M, Shapiro R, Yang R, Noehren B (2014). Accuracy and repeatability of joint angles measured using a single camera markerless motion capture system. J Biomech.

[24] Shotton J, Sharp T, Kipman A, Fitzgibbon A, Finocchio M, Blake A (2013). Real-time human pose recognition in parts from single depth images. Commun ACM.

[25] Zhang Z (2012). Microsoft kinect sensor and its effect. IEEE Multimed.

[26] Han J, Shao L, Xu D, Shotton J (2013). Enhanced computer vision with microsoft kinect sensor: a review. IEEE Trans Cybern.

[27] Mousavi Hondori H, Khademi M (2014). A review on technical and clinical impact of microsoft kinect on physical therapy and rehabilitation. J Med Eng.

[28] Yang L, Zhang L, Dong H, Alelaiwi A, El Saddik A (2015). Evaluating and improving the depth accuracy of kinect for Windows v2. IEEE Sens J.

[29] Capecci M, Ceravolo M, Ferracuti F, Iarlori S, Longhi S, Romeo L, et al. Accuracy evaluation of the Kinect v2 sensor during dynamic movements in a rehabilitation scenario. In: Intl. Conf. of the Eng. in Medicine and Biology Society (EMBC). IEEE; 2016. p. 5409–12. 10.1109/EMBC.2016.7591950 28269481

[30] Corti A, Giancola S, Mainetti G, Sala R (2016). A metrological characterization of the kinect V2 time-of-flight camera. Robot Autonom Syst.

[31] Sarbolandi H, Lefloch D, Kolb A (2015). Kinect range sensing: structured-light versus time-of-flight kinect. Comput Vis Image Underst.

[32] Pagliari D, Pinto L (2015). Calibration of kinect for xbox one and comparison between the two generations of Microsoft sensors. Sensors.

[33] Mortazavi F, Nadian-Ghomsheh A (2018). Stability of Kinect for range of motion analysis in static stretching exercises. PLoS ONE.

[34] Pedro LM, de Paula Caurin GA. Kinect evaluation for human body movement analysis. In: Biomedical robotics and biomechatronics (BioRob). IEEE; 2012. p. 1856–61.

[35] Da Gama A, Fallavollita P, Teichrieb V, Navab N (2015). Motor rehabilitation using kinect: a systematic review. Games Health J.

[36] Clark RA, Pua YH, Oliveira CC, Bower KJ, Thilarajah S, McGaw R (2015). Reliability and concurrent validity of the Microsoft Xbox one kinect for assessment of standing balance and postural control. Gait Posture.

[37] Grooten WJA, Sandberg L, Ressman J, Diamantoglou N, Johansson E, Rasmussen-Barr E (2018). Reliability and validity of a novel kinect-based software program for measuring posture, balance and side-bending. BMC Musculoskelet Disord.

[38] Tran TH, Le TL, Hoang VN, Vu H (2017). Continuous detection of human fall using multimodal features from kinect sensors in scalable environment. Comput Methods Prog Biomed.

[39] Kuster RP, Heinlein B, Bauer CM, Graf ES (2016). Accuracy of kinectone to quantify kinematics of the upper body. Gait Posture.

[40] Otte K, Kayser B, Mansow-Model S, Verrel J, Paul F, Brandt AU (2016). Accuracy and reliability of the kinect version 2 for clinical measurement of motor function. PLoS ONE.

[41] Ma M, Proffitt R, Skubic M (2018). Validation of a kinect V2 based rehabilitation game. PLoS ONE.

[42] Mentiplay BF, Perraton LG, Bower KJ, Pua YH, McGaw R, Heywood S (2015). Gait assessment using the Microsoft Xbox one kinect: concurrent validity and inter-day reliability of spatiotemporal and kinematic variables. J Biomech.

[43] Dolatabadi E, Taati B, Mihailidis A (2016). Concurrent validity of the Microsoft Kinect for Windows v2 for measuring spatiotemporal gait parameters. Med Eng Phys.

[44] Müller B, Ilg W, Giese MA, Ludolph N (2017). Validation of enhanced kinect sensor based motion capturing for gait assessment. PLoS ONE.

[45] Valdés BA, Hilderman CG, Hung CT, Shirzad N, Van der Loos HM. Usability testing of gaming and social media applications for stroke and cerebral palsy upper limb rehabilitation. In: 36th annual international conference of the IEEE engineering in medicine and biology society. IEEE. 2014;2014:3602–5. 10.1109/EMBC.2014.6944402 25570770

[46] Brokaw EB, Eckel E, Brewer BR (2015). Usability evaluation of a kinematics focused kinect therapy program for individuals with stroke. Technol Health Care.

[47] Xu X, McGorry RW (2015). The validity of the first and second generation Microsoft Kinect$$^{{rm TM}}$$ for identifying joint center locations during static postures. Appl Ergon.

[48] Wang Q, Kurillo G, Ofli F, Bajcsy R. Evaluation of pose tracking accuracy in the first and second generations of microsoft kinect. In: Intl. Conf. on healthcare informatics (ICHI). IEEE; 2015. p. 380–9.

[49] Obdržálek Š, Kurillo G, Ofli F, Bajcsy R, Seto E, Jimison H, et al. Accuracy and robustness of Kinect pose estimation in the context of coaching of elderly population. In: Intl. Conf of the IEEE engineering in medicine and biology society (EMBS). IEEE; 2012. p. 1188–93. 10.1109/EMBC.2012.6346149 23366110

[50] Clark RA, Pua YH, Fortin K, Ritchie C, Webster KE, Denehy L (2012). Validity of the Microsoft Kinect for assessment of postural control. Gait Posture.

[51] Bonnechere B, Jansen B, Salvia P, Bouzahouene H, Omelina L, Moiseev F (2014). Validity and reliability of the kinect within functional assessment activities: comparison with standard stereophotogrammetry. Gait Posture.

[52] Galna B, Barry G, Jackson D, Mhiripiri D, Olivier P, Rochester L (2014). Accuracy of the Microsoft Kinect sensor for measuring movement in people with Parkinson’s disease. Gait Posture.

[53] van Diest M, Stegenga J, Wörtche HJ, Postema K, Verkerke GJ, Lamoth CJ (2014). Suitability of kinect for measuring whole body movement patterns during exergaming. J Biomech.

[54] Schmitz A, Ye M, Boggess G, Shapiro R, Yang R, Noehren B (2015). The measurement of in vivo joint angles during a squat using a single camera markerless motion capture system as compared to a marker based system. Gait Posture.

[55] Takeda R, Tadano S, Natorigawa A, Todoh M, Yoshinari S (2009). Gait posture estimation using wearable acceleration and gyro sensors. J Biomech.

[56] Cutti AG, Ferrari A, Garofalo P, Raggi M, Cappello A, Ferrari A (2010). Outwalk: a protocol for clinical gait analysis based on inertial and magnetic sensors. Med Biol Eng Comput.

[57] Ahmadi A, Mitchell E, Richter C, Destelle F, Gowing M, O’Connor NE (2015). Toward automatic activity classification and movement assessment during a sports training session. IEEE Internet Things J.

[58] Giggins OM, Sweeney KT, Caulfield B (2014). Rehabilitation exercise assessment using inertial sensors: a cross-sectional analytical study. J Neuroeng Rehabil.

[59] Bergamini E, Ligorio G, Summa A, Vannozzi G, Cappozzo A, Sabatini AM (2014). Estimating orientation using magnetic and inertial sensors and different sensor fusion approaches: accuracy assessment in manual and locomotion tasks. Sensors.

[60] Seel T, Raisch J, Schauer T (2014). IMU-based joint angle measurement for gait analysis. Sensors.

[61] Fantozzi S, Giovanardi A, Magalhães FA, Di Michele R, Cortesi M, Gatta G (2016). Assessment of three-dimensional joint kinematics of the upper limb during simulated swimming using wearable inertial-magnetic measurement units. J Sports Sci.

[62] Papi E, Osei-Kuffour D, Chen YMA, McGregor AH (2015). Use of wearable technology for performance assessment: a validation study. Med Eng Phys.

[63] Lebel K, Boissy P, Nguyen H, Duval C (2017). Inertial measurement systems for segments and joints kinematics assessment: towards an understanding of the variations in sensors accuracy. Biomed Eng OnLine.

[64] Chiang CY, Chen KH, Liu KC, Hsu SJP, Chan CT (2017). Data collection and analysis using wearable sensors for monitoring knee range of motion after total knee arthroplasty. Sensors.

[65] Schall MC, Fethke NB, Chen H, Oyama S, Douphrate DI (2015). Accuracy and repeatability of an inertial measurement unit system for field-based occupational studies. Ergonomics.

[66] Tian Y, Meng X, Tao D, Liu D, Feng C (2015). Upper limb motion tracking with the integration of IMU and kinect. Neurocomputing.

[67] Destelle F, Ahmadi A, O’Connor NE, Moran K, Chatzitofis A, Zarpalas D, et al. Low-cost accurate skeleton tracking based on fusion of kinect and wearable inertial sensors. In: 22nd European signal processing conference (EUSIPCO). IEEE; 2014. p. 371–5.

[68] Kyrarini M, Wang X, Gräser A. Comparison of vision-based and sensor-based systems for joint angle gait analysis. In: IEEE intl. symp. on medical measurements and applications (MeMeA). IEEE; 2015. p. 375–9.

[69] Yang GZ, Yacoub M (2006). Body sensor networks.

[70] Hanson MA, Powell HC, Barth AT, Ringgenberg K, Calhoun BH, Aylor JH (2009). Body area sensor networks: challenges and opportunities. Computer.

[71] Roetenberg D, Luinge H, Slycke P. Xsens MVN: full 6DOF human motion tracking using miniature inertial sensors. Xsens Motion Technologies BV, technical report; 2009.

[72] Lane ND, Miluzzo E, Lu H, Peebles D, Choudhury T, Campbell AT (2010). A survey of mobile phone sensing. Commun Mag IEEE.

[73] Burns A, Greene BR, McGrath MJ, O’Shea TJ, Kuris B, Ayer SM (2010). SHIMMER—a wireless sensor platform for noninvasive biomedical research. Sens J IEEE.

[74] Harms H, Amft O, Winkler R, Schumm J, Kusserow M, Tröster G. Ethos: miniature orientation sensor for wearable human motion analysis. In: IEEE sensors. IEEE; 2010. p. 1037–42.

[75] Brigante C, Abbate N, Basile A, Faulisi AC, Sessa S (2011). Towards miniaturization of a MEMS-based wearable motion capture system. IEEE Trans Ind Electron.

[76] Bruckner HP, Nowosielski R, Kluge H, Blume H. Mobile and wireless inertial sensor platform for motion capturing in stroke rehabilitation sessions. In: IEEE intl. workshop on advances in sensors and interfaces (IWASI). IEEE; 2013. p. 14–9.

[77] Comotti D, Ermidoro M, Galizzi M, Vitali AL. Development of a wireless low-power multi-sensor network for motion tracking applications. In: Intl. conf. on wearable and implantable body sensor networks (BSN). IEEE; 2013. p. 1–6.

[78] Rodríguez-Martín D, Pérez-López C, Samà A, Cabestany J, Català A (2013). A wearable inertial measurement unit for long-term monitoring in the dependency care area. Sensors.

[79] Sabatini AM (2011). Estimating three-dimensional orientation of human body parts by inertial/magnetic sensing. Sensors.

[80] Bugané F, Benedetti M, Casadio G, Attala S, Biagi F, Manca M (2012). Estimation of spatial-temporal gait parameters in level walking based on a single accelerometer: validation on normal subjects by standard gait analysis. Comput Methods Prog Biomed.

[81] Young AD. Comparison of orientation filter algorithms for realtime wireless inertial posture tracking. In: Intl. workshop on wearable and implantable body sensor networks (BSN). IEEE; 2009. p. 59–4.

[82] Sabatini AM. Inertial sensing in biomechanics: a survey of computational techniques bridging motion analysis and personal navigation. In: Computational intelligence for movement sciences: neural networks and other emerging techniques. IGI Global; 2006. p. 70–100.

[83] Mahony R, Hamel T, Pflimlin JM (2008). Nonlinear complementary filters on the special orthogonal group. IEEE Trans Autom Control.

[84] Madgwick SO, Harrison AJ, Vaidyanathan R. Estimation of IMU and MARG orientation using a gradient descent algorithm. In: IEEE intl. conf. on rehabilitation robotics (ICORR). IEEE; 2011. p. 1–7. 10.1109/ICORR.2011.5975346 22275550

[85] Bulling A, Blanke U, Schiele B (2014). A tutorial on human activity recognition using body-worn inertial sensors. ACM Comput Surv (CSUR).

[86] Morris D, Saponas TS, Guillory A, Kelner I. RecoFit: using a wearable sensor to find, recognize, and count repetitive exercises. In: Proceedings of the 32nd annual ACM conference on Human factors in computing systems. ACM; 2014. p. 3225–34.

[87] Altini M, Penders J, Vullers R, Amft O (2015). Estimating energy expenditure using body-worn accelerometers: a comparison of methods, sensors number and positioning. IEEE J Biomed Health Inform.

[88] Casamassima F, Ferrari A, Milosevic B, Ginis P, Farella E, Rocchi L (2014). A wearable system for gait training in subjects with Parkinson’s disease. Sensors.

[89] Ferrari A, Ginis P, Hardegger M, Casamassima F, Rocchi L, Chiari L (2016). A mobile Kalman-filter based solution for the real-time estimation of spatio-temporal gait parameters. IEEE Trans Neural Syst Rehabil Eng.

[90] Picerno P, Camomilla V, Capranica L (2011). Countermovement jump performance assessment using a wearable 3D inertial measurement unit. J Sports Sci.

[91] Milosevic B, Farella E. Wearable Inertial Sensor for Jump Performance Analysis. In: Proc. of the 2015 workshop on wearable systems and applications (WearSys). ACM; 2015. p. 15–20.

[92] Buganè F, Benedetti MG, D’Angeli V, Leardini A (2014). Estimation of pelvis kinematics in level walking based on a single inertial sensor positioned close to the sacrum: validation on healthy subjects with stereophotogrammetric system. Biomed Eng OnLine.

[93] Hubble RP, Naughton GA, Silburn PA, Cole MH (2015). Wearable sensor use for assessing standing balance and walking stability in people with Parkinson’s disease: a systematic review. PLoS ONE.

[94] Cui J, Chen J, Qu G, Starkman J, Zeng X, Madigan E (2017). Wearable Gait Lab System providing quantitative statistical support for human balance tests. Smart Health.

[95] Bagalà F, Becker C, Cappello A, Chiari L, Aminian K, Hausdorff JM (2012). Evaluation of accelerometer-based fall detection algorithms on real-world falls. PLoS ONE.

[96] Ferrari A, Cutti AG, Garofalo P, Raggi M, Heijboer M, Cappello A (2010). First in vivo assessment of “Outwalk”: a novel protocol for clinical gait analysis based on inertial and magnetic sensors. Med Biol Eng Comput.

[97] Palmerini L, Mellone S, Avanzolini G, Valzania F, Chiari L (2013). Quantification of motor impairment in Parkinson’s disease using an instrumented timed up and go test. IEEE Trans Neural Syst Rehabil Eng.

[98] Zhao Z, Etemad SA, Arya A, Whitehead A. Usability and motivational effects of a gamified exercise and fitness system based on wearable devices. In: International conference of design, user experience, and usability. Springer; 2016. p. 333–44.

[99] Zhao Z, Arya A, Whitehead A, Chan G, Etemad SA. Keeping users engaged through feature updates: a long-term study of using wearable-based exergames. In: Conference on human factors in computing systems (CHI); 2017. p. 1053–64.

[100] Wang Q, Markopoulos P, Yu B, Chen W, Timmermans A (2017). Interactive wearable systems for upper body rehabilitation: a systematic review. J Neuroeng Rehabil.

[101] Glonek G, Wojciechowski A. Kinect and IMU sensors imprecisions compensation method for human limbs tracking. In: Intl. conf. on computer vision and graphics. Springer; 2016. p. 316–28.

[102] Haggag H, Hossny M, Nahavandi S, Haggag O. An adaptable system for rgb-d based human body detection and pose estimation: Incorporating attached props. In: IEEE int. conf. on systems, man, and cybernetics (SMC); 2016. p. 001544–9.

[103] Shcheglov K, Evans C, Gutierrez R, Tang TK. Temperature dependent characteristics of the JPL silicon MEMS gyroscope. In: Aerospace conference proceedings. vol. 1. IEEE; 2000. p. 403–11.

[104] Wen M, Wang W, Luo Z, Xu Y, Wu X, Hou F, et al. Modeling and analysis of temperature effect on MEMS gyroscope. In: Electronic components and technology conference (ECTC), 2014 IEEE 64th. IEEE; 2014. p. 2048–52.

[105] Farrell J (2008). Aided navigation: GPS with high rate sensors.

[106] Kekade S, Hseieh CH, Islam MM, Atique S, Khalfan AM, Li YC (2018). The usefulness and actual use of wearable devices among the elderly population. Comput Methods Prog Biomed.

[107] Hiremath S, Yang G, Mankodiya K. Wearable Internet of Things: Concept, architectural components and promises for person-centered healthcare. In: Intl. conf. on wireless mobile communication and healthcare (Mobihealth). IEEE; 2014. p. 304–7.

[108] Webster D, Celik O (2014). Systematic review of kinect applications in elderly care and stroke rehabilitation. J Neuroeng Rehabil.

[109] Chen H, Schall MC, Fethke N (2018). Accuracy of angular displacements and velocities from inertial-based inclinometers. Appl Ergon.

[110] McGinley JL, Baker R, Wolfe R, Morris ME (2009). The reliability of three-dimensional kinematic gait measurements: a systematic review. Gait Posture.

[111] Ricci L, Taffoni F, Formica D (2016). On the orientation error of IMU: investigating static and dynamic accuracy targeting human motion. PLoS ONE.

[112] Roell M, Roecker K, Gehring D, Mahler H, Gollhofer A (2018). Player monitoring in indoor team sports: concurrent validity of inertial measurement units to quantify average and peak acceleration values. Front Physiol.

[113] Leardini A, Sawacha Z, Paolini G, Ingrosso S, Nativo R, Benedetti MG (2007). A new anatomically based protocol for gait analysis in children. Gait Posture.

[114] Wu G, Siegler S, Allard P, Kirtley C, Leardini A, Rosenbaum D (2002). ISB recommendation on definitions of joint coordinate system of various joints for the reporting of human joint motion-part I: ankle, hip, and spine. J Biomech.

[115] Moran RW, Schneiders AG, Major KM, Sullivan SJ (2016). How reliable are functional movement screening scores? A systematic review of rater reliability. Br J Sports Med.

[116] Nae J, Creaby MW, Nilsson G, Crossley KM, Ageberg E (2017). Measurement properties of a test battery to assess postural orientation during functional tasks in patients undergoing anterior cruciate ligament injury rehabilitation. J Orthopaedic Sports Phys Ther.

